# Attainment of clinical performance targets and improvement in clinical outcomes and resource use in hemodialysis care: a prospective cohort study

**DOI:** 10.1186/1472-6963-7-5

**Published:** 2007-01-09

**Authors:** Laura C Plantinga, Nancy E Fink, Bernard G Jaar, John H Sadler, Nathan W Levin, Josef Coresh, Michael J Klag, Neil R Powe

**Affiliations:** 1 Department of Medicine, Johns Hopkins University School of Medicine, Baltimore, MD21205, USA; 2 Department of Epidemiology, Johns Hopkins Bloomberg School of Public Health, Baltimore, MD21205, USA; 3 Independent Dialysis Foundation, Baltimore, MD21201, USA; 4 Renal Research Institute, New York, NY10128, USA; 5 Department of Biostatistics, Johns Hopkins Bloomberg School of Public Health, Baltimore, MD21205, USA; 6 Department of Health Policy and Management, Johns Hopkins Bloomberg School of Public Health, Baltimore, MD21205, USA

## Abstract

**Background:**

Clinical performance targets are intended to improve patient outcomes in chronic disease through quality improvement, but evidence of an association between multiple target attainment and patient outcomes in routine clinical practice is often lacking.

**Methods:**

In a national prospective cohort study (ESRD Quality, or EQUAL), we examined whether attainment of multiple targets in 668 incident hemodialysis patients from 74 U.S. not-for-profit dialysis clinics was associated with better outcomes. We measured whether the following accepted clinical performance targets were met at 6 months after study enrollment: albumin (≥4.0 g/dl), hemoglobin (≥11 g/dl), calcium-phosphate product (<55 mg^2^/dl^2^), dialysis dose (Kt/V≥1.2), and vascular access type (fistula). Outcomes included mortality, hospital admissions, hospital days, and hospital costs.

**Results:**

Attainment of each of the five targets was associated individually with better outcomes; *e.g*., patients who attained the albumin target had decreased mortality [relative hazard (RH) = 0.55, 95% confidence interval (CI), 0.41–0.75], hospital admissions [incidence rate ratio (IRR) = 0.67, 95% CI, 0.62–0.73], hospital days (IRR = 0.61, 95% CI, 0.58–0.63), and hospital costs (average annual cost reduction = $3,282, *P *= 0.002), relative to those who did not. Increasing numbers of targets attained were also associated, in a graded fashion, with decreased mortality (*P *= 0.030), fewer hospital admissions and days (*P *< 0.001 for both), and lower costs (*P *= 0.029); these trends remained statistically significant for all outcomes after adjustment (*P *< 0.001), except cost, which was marginally significant (*P *= 0.052).

**Conclusion:**

Attainment of more clinical performance targets, regardless of which targets, was strongly associated with decreased mortality, hospital admissions, and resource use in hemodialysis patients.

## Background

Both improvement in the quality of delivered care and its measurement are of considerable interest to healthcare providers and researchers [1-3]. In particular, for those providing care to kidney disease patients, the National Kidney Foundation began the Kidney Disease Dialysis Outcomes Quality Initiative (KDOQI) [4] in 1995, "in keeping with its longstanding commitment to improving the quality of care delivered to all patients with kidney disease and the firm conviction that substantial improvements in the quality and outcomes of their care are achievable." This program has published several clinical performance guidelines [4], based in part upon evidence of improved outcomes and in part upon expert judgment, that address aspects of end-stage renal disease (ESRD) patient care related to nutrition, bone disease management, anemia management, adequacy of dialysis, and vascular access placement.

Higher serum albumin, a marker of better nutrition and lower inflammatory burden, has been well-established as a predictor of better survival in dialysis patients [5-10]. Lower levels of calcium-phosphate (Ca-P) product, a marker of effective bone disease management, have also been shown to be associated with decreased mortality [11]. Renal anemia, reflected by low hemoglobin levels [12-14], and low dialysis doses [15-18] have been associated with poor outcomes, which are fewer with the placement of a native arteriovenous fistula versus a graft or catheter [19,20]. It has also been suggested that bringing hemodialysis patients within guidelines for multiple clinical performance targets may confer greater survival [21].

These guidelines have led to improvement in the process of kidney disease care, as evidenced by improvement in clinical performance measures, and have even led to calls for financial incentives for further improvement [22]. However, whether attainment of accepted clinical performance targets for nutrition, bone disease management, anemia management, dialysis adequacy, and vascular access placement by hemodialysis patients have led to fewer hospitalizations, fewer hospital days, or decreased hospitalization costs, in addition to better patient survival, has been less well-studied [23,24]. In addition, the attainment of multiple clinical performance targets may or may not confer greater protection than the attainment of any single target. Thus, we conducted a national study to examine whether both individual and multiple clinical performance target attainment in end-stage renal disease patients was associated with improvement in these clinical outcomes.

## Methods

### Study design

The ESRD Quality (EQUAL) study was designed to measure processes of care and outcomes in the Choices for Healthy Outcomes in Caring for End-Stage Renal Disease (CHOICE) [25] cohort. The population examined here consisted of 668 incident hemodialysis patients who were treated at 74 U.S. not-for-profit, free-standing outpatient dialysis clinics in 18 states. To be included in this study, patients had to be treated with hemodialysis and survive at least 6 months. The CHOICE cohort consisted of 1041 incident dialysis patients (767 hemodialysis, 274 peritoneal dialysis) enrolled in a national observational study of treatment effectiveness study at 81 dialysis clinics in 19 states between October 1995 and June 1998. Neither the CHOICE nor the EQUAL study involved the use of any intervention. To be eligible, patients had to be older than 18 years of age and speak either English or Spanish. Median time from dialysis initiation to enrollment was 45 days, with 98% enrolling within 4 months of initial dialysis. Informed consent was obtained from each patient. Institutional review boards for the Johns Hopkins University School of Medicine and clinical centers approved the study protocol.

### Data collection

Measures of whether patients attained clinical performance targets at 6 months after enrollment in the study served as the independent variables for this study. The clinical performance measures examined included 6-month values for albumin, hemoglobin, Ca-P product, and dialysis dose and ascertainment of the type of vascular access in use at 6 months. Albumin, hemoglobin, calcium, and phosphate levels were obtained from routine laboratory data. All serum albumin levels were measured at a central laboratory using the Bromocresol Green method (CV, 1.1%). Calcium levels were corrected for albumin [corrected calcium = calcium level + 0.8*(4-albumin level)] before Ca-P products were calculated. Vascular access information was obtained on a subset of individuals through review of discharge summaries, dialysis flow sheets, and dialysis clinic progress notes [26]. Dialysis dose (Kt/V) was calculated from values of blood urea nitrogen, pre- and post-dialysis, weight, and dialysis duration using the Daugirdas formula [27]. The targets we selected for these measures, which were based upon the KDOQI Clinical Practice Guidelines [4], were: albumin, ≥4.0 g/dl; hemoglobin, ≥11 g/dl (based upon the lower limit of the guideline range of 11–12 g/dl); Ca-P product, <55 mg^2^/dl^2^; dialysis dose, Kt/V≥1.2; and vascular access type, presence of functioning arteriovenous fistula. In addition to individual targets, the total number of targets attained was also used as an independent variable. For total number of targets attained, data were collapsed into four categories (zero or one target, two targets, three targets, and four or five targets attained). The numbers of patients attaining no (*n *= 3) or all five targets (*n *= 17) were small. We also examined a subset of targets (albumin, Ca-P product, and hemoglobin) that are regularly measured as part of the usual laboratory panels.

Outcome variables included all-cause mortality, number of hospitalizations, days hospitalized, and hospitalization costs. Mortality information was ascertained from clinic report, medical records, and Centers for Medicare & Medicaid Services (CMS; death notification forms and Social Security records). Follow-up continued until death, transplantation, loss to follow-up, or the last follow-up date of November 2003. Hospitalization data were obtained through the United States Renal Data System (USRDS) [28] and were available through November 2001. Hospitalization costs were defined as the amount paid by Medicare for hospitalizations; for hospitalizations that were paid by another insurer, a payment-to-charge ratio, calculated from those records exclusively paid by Medicare, was applied to the charges to estimate the Medicare payment.

We also collected data on demographic, laboratory, and clinical characteristics. Data regarding patient demographics (age, sex, and race) and socioeconomic status (education and employment) were collected from a baseline self-report questionnaire. Laboratory values and height and weight [used to calculate body mass index (BMI)] were obtained from clinic records and from the CMS Medical Evidence report (CMS Form 2728). Comorbidity was assessed at baseline using the Index of Coexistent Disease (ICED), a validated measure whose composite integer score ranges from 0 to 3 (with 3 as the highest severity level) and is a measure of both the presence and severity of comorbid conditions [29-32]. The ICED is derived from the peak scores of the Index of Disease Severity and the Index of Physical Impairment using an algorithm specific to the ICED. The Index of Disease Severity consists of 19 categories of medical conditions, with four levels of severity for each condition. Information for the IDS was abstracted from dialysis unit records, hospital discharge summaries, medication lists, consultation notes, diagnostic imaging, and cardiac imaging reports. Two dialysis nurses, with prior training and experience in using the ICED, reviewed and scored all charts. The Index of Physical Impairment is an observer-based assessment of 11 functional domains, each with three severity levels, completed by a local dialysis nurse familiar with the patient's level of functioning, with input from a family member or caregiver, if necessary. Late referral was defined as less than 4 months between first nephrologist evaluation and start of dialysis [33]. Frequency of physician contact and sit-down rounds were as described previously [34,35]; noncompliance with dialysis was defined as missing >3% of hemodialysis sessions.

### Statistical methods

We first compared patient characteristics by whether patients attained individual targets and by the total number of targets they attained using Pearson's χ^2 ^tests for categorical variables and two-sided *t *tests or ANOVA for continuous variables. Observed survival time was assessed starting at time of patient enrollment and ending at death or censoring (at transplant, loss to follow-up, or closeout), and we assessed individual cumulative mortality by whether patients attained targets by calculating overall crude mortality rates at the average follow-up (2.8 years) and by using Kaplan-Meier methods. We used Cox proportional hazards models to assess the strength and independence of an association between target attainment and survival. The proportional hazards assumption was not violated for any of the targets or for the total number of targets attained (*p *> 0.05 by global test of Schoenfeld residuals). Crude hospitalization rates were calculated, and Poisson regression models were used to assess the relation between target attainment and hospitalization (incidence rate ratio). Linear regression was used to assess the association between attainment of targets and hospitalization (after logarithmic transformation) costs per year. For all regression models, attainment of individual targets, the subset of three laboratory targets, and all five targets were examined.

Variables were considered for adjustment in the multivariable regression models based on either their demonstration to be confounders (*i.e*., significantly associated with both target attainment and the outcome measure) or prior evidence of their association with the outcome. Adjustment for both baseline target values (the major confounders) and other confounders of the outcome was performed. We also used models incorporating both target attainment and propensity scores for target attainment. Propensity score modeling is an established technique used to address selection bias [36]. For multiple targets, attainment was dichotomized for the three laboratory targets (two or three targets versus zero or one) and for all five targets (three or more targets attained versus two or fewer), and propensity scores were obtained similarly. Sensitivity analyses examining only those patients who had not attained the individual target in question at baseline and also those who had attained either no targets or only one target at baseline were also performed. We accounted for possible within-clinic correlation by using conditional methods (fixed effects or stratification by clinic) in all models presented. Likelihood ratio tests were performed to determine each individual target's contribution to variation in the outcome of interest. Statistical significance was set at P < 0.05. However, because examining five targets in a small sample might lead to randomly significant results at this cutoff, we decided to also set a Bonferroni-correct threshold *P *value of 0.05/5 = 0.01 for the individual target analyses. All analyses were performed using STATA v. 8.2 (College Station, Texas).

## Results

### Patient characteristics by attainment of targets

Relative to patients not attaining 6-month targets (Table 1), (*i*) albumin target attainers were younger, more likely to be male, and less likely to have a fistula at the start of hemodialysis, and had lower comorbidity scores, higher baseline albumin and creatinine, and lower initial CRP; (*ii*) Ca-P target attainers were older and less likely to be employed or noncompliant with dialysis and had higher comorbidity scores, higher Kt/V, lower creatinine, higher hemoglobin, and lower initial Ca-P product; (*iii*) hemoglobin target attainers were older and more likely to be male and white and had less frequent physician visits, lower BMI, higher Kt/V, and higher initial hemoglobin; (*iv*) dialysis dose target attainers were older and less likely to be male and had more frequent physician visits lower BMI, lower creatinine, higher hemoglobin, lower Ca-P, and higher initial dose (Kt/V); and (*v*) access target attainers were younger and more likely to be male and white and had lower dose, higher albumin, lower CRP, and fewer fistulas (none) at start of dialysis, relative to those not attaining the target. For those who were at or above targets at 6 months, 26.1%, 81.2%, 23.2%, 61.5%, and 56.5% of patients were already at the albumin, Ca-P product, hemoglobin, dialysis dose, and vascular access targets, respectively, at baseline. Finally, those patients attaining a greater number of targets at 6 months (Table 1) had lower comorbidity, BMI, and CRP; and were more compliant with dialysis and generally closer to the targets at baseline than those who attained fewer targets (data not shown). For those with information on all five targets (*n *= 344) at 6 months, 39% (*n *= 133) had attained 0 or 1 target at baseline; 41%, 16%, and 4% of these patients had attained 2, 3, and 4 or 5 targets at baseline, respectively.

**Table 1:** Baseline patient characteristics by attainment of 6-month clinical performance targets

**Characteristic**	**Albumin Target Attained (N = 650)**			**Ca-P Product Target Attained (N = 648)**			**Hemoglobin Target Attained (N = 644)**			**Dialysis Dose Target Attained (N = 539)**			**Access Target Attained (N = 425)**			**Total Number of Targets Attained (N = 344)**				
	
	**No**	**Yes**	P*	**No**	**Yes**	P*	**No**	**Yes**	P*	**No**	**Yes**	P*	**No**	**Yes**	P*	**0–1**	**2**	**3**	**4–5**	P*
N (%)	451 (69)	199 (31)	--	250 (39)	398 (61)	--	256 (40)	388 (60)	--	95 (18)	444 (82)	--	310 (73)	115 (27)	--	40 (12)	116 (33)	117 (34)	71 (21)	--
**Demographic**																				
Mean age	60.4 ± 13.6	56.9 ± 15.7	**0.005**	56.3 ± 13.8	61.1 ± 14.3	**<0.001**	57.4 ± 14.5	60.4 ± 14.0	**0.015**	55.1 ± 14.3	60.6 ± 14.1	**<0.001**	60.1 ± 14.5	56.1 ± 13.5	**0.010**	58.4 ± 12.8	58.4 ± 15.2	61.6 ± 13.3	58.4 ± 13.9	0.255
Sex (% male)	46.8	66.8	**<0.001**	57.6	50.0	0.059	46.9	56.7	**0.015**	74.7	48.7	**<0.001**	47.4	69.6	**<0.001**	57.5	43.1	51.3	74.7	**<0.001**
Race (% white)	59.0	64.8	0.320	65.2	58.3	0.122	55.1	64.4	**0.042**	61.1	58.1	0.806	57.4	68.7	**0.001**	65.0	56.0	55.6	67.6	0.461
Education (% HS grad)	65.3	72.3	0.084	68.9	66.5	0.538	65.4	68.7	0.379	67.7	66.1	0.765	67.9	67.9	0.998	65.0	62.9	68.8	70.6	0.690
Employment (% employed)	8.4	9.6	0.628	12.0	6.8	**0.023**	7.8	9.6	0.445	10.5	8.1	0.448	8.7	11.4	0.400	10.0	10.3	9.4	10.0	0.996
**Clinical**																				
Access (% fistula)	23.3	11.9	**0.007**	30.8	30.2	0.943	23.9	17.8	0.120	15.5	21.8	0.283	29.0	0.0	**<0.001**	27.5	26.7	25.6	5.6	**0.003**
Diabetes (% diabetic)	60.5	44.7	**<0.001**	56.4	55.0	0.732	59.8	52.8	0.083	62.1	55.6	0.248	59.7	47.8	**0.029**	62.5	62.1	63.3	39.4	**0.006**
ICED (% with score of 3)	33.7	22.6	**0.003**	26.1	37.1	**0.008**	33.2	28.9	0.503	33.7	31.1	0.151	33.6	21.7	**<0.001**	37.5	41.4	26.5	23.9	**0.027**
Late referral (% <4mo)	33.6	31.2	0.577	17.7	21.6	0.322	32.6	32.9	0.931	40.0	32.7	0.212	34.7	16.7	**0.001**	34.5	30.0	31.6	26.2	0.848
Mean BMI	27.7 ± 7.3	26.8 ± 6.3	0.144	28.1 ± 7.1	27.1 ± 6.9	0.061	28.6 ± 8.1	26.7 ± 6.0	**0.001**	30.5 ± 8.4	27.1 ± 6.6	**<0.001**	27.5 ± 7.2	27.9 ± 6.5	0.593	31.5 ± 9.8	27.7 ± 6.8	28.4 ± 6.4	25.9 ± 5.8	**0.001**
Physician visit (% weekly)	14.5	14.3	0.995	13.9	14.8	0.605	19.6	11.2	**0.013**	9.8	17.0	**<0.001**	15.0	14.3	0.663	24.6	14.1	12.3	14.8	0.213
Sit-down rounds (% monthly)	40.6	47.6	0.103	45.8	40.5	0.195	39.6	43.9	0.294	38.0	42.0	0.487	38.5	41.1	0.639	36.1	44.5	38.6	48.2	0.280
Dialysis compliant (% noncompliant)	9.4	6.4	0.211	11.4	6.7	**0.040**	10.8	7.1	0.102	11.6	7.0	0.134	9.0	5.2	0.198	9.0	12.4	6.6	1.2	**0.008**
**Laboratory**																				
Mean Kt/V	1.26 ± 0.35	1.26 ± 0.29	0.910	1.21 ± 0.33	1.28 ± 0.33	**0.010**	1.22 ± 0.33	1.28 ± 0.33	**0.048**	0.99 ± 0.28	1.29 ± 0.31	**<0.001**	1.30 ± 0.33	1.19 ± 0.36	**0.006**	1.09 ± 0.31	1.29 ± 0.33	1.20 ± 0.35	1.30 ± 0.30	**0.002**
Mean albumin	3.41 ± 0.45	3.68 ± 0.44	**<0.001**	3.47 ± 0.42	3.51 ± 0.49	0.359	3.45 ± 0.49	3.52 ± 0.44	0.074	3.43 ± 0.50	3.52 ± 0.45	0.107	3.46 ± 0.46	3.60 ± 0.42	**0.003**	3.42 ± 0.47	3.42 ± 0.50	3.51 ± 0.38	3.70 ± 0.43	**<0.001**
Mean creatinine	6.88 ± 2.20	8.37 ± 2.69	**<0.001**	7.85 ± 2.59	7.01 ± 2.32	**<0.001**	7.51 ± 2.42	7.20 ± 2.48	0.115	7.80 ± 2.49	7.17 ± 2.40	**0.022**	6.94 ± 2.26	7.73 ± 2.47	**0.002**	7.48 ± 1.96	6.83 ± 2.37	7.21 ± 2.40	7.24 ± 2.32	0.384
Mean hemoglobin	9.74 ± 1.45	9.86 ± 1.60	0.337	9.59 ± 1.46	9.89 ± 1.50	**0.012**	9.50 ± 1.41	9.95 ± 1.52	**<0.001**	9.46 ± 1.50	9.88 ± 1.50	**0.013**	9.73 ± 1.39	9.80 ± 1.50	0.621	9.54 ± 1.33	9.60 ± 1.35	9.71 ± 1.44	10.2 ± 1.46	**0.022**
Mean Ca-P product	45.8 ± 14.3	47.9 ± 14.4	0.091	50.8 ± 14.6	43.8 ± 13.5	**<0.001**	46.9 ± 15.2	46.2 ± 13.7	0.516	48.8 ± 14.3	45.6 ± 14.1	**0.043**	46.2 ± 14.9	47.6 ± 13.6	0.388	53.8 ± 15.8	44.4 ± 14.2	46.9 ± 14.5	43.5 ± 12.0	**0.001**
Median CRP (IQR)	4.4(2.1,12.8)	3.4 (1.3,5.5)	**<0.001**	3.9(1.6,12.0)	4.0 (1.8,9.4)	0.461	4.3 (2.1,13)	3.8 (1.7,7.5)	0.482	4.6 (2.6,14)	3.8 (1.6,9.4)	0.942	3.9 (2.0,12)	3.4 (1.4,5.8)	**0.035**	5.5 (3.5,14.7)	3.5 (1.6,10.6)	3.6 (1.9,6.5)	3.6 (1.3,5.4)	**0.020**
Median ferritin (IQR)	118 (63,261)	143 (67,272)	0.572	117 (66,268)	142 (63,265)	0.312	154 (70,317)	119 (62,247)	0.078	118 (62,266)	133 (65,260)	0.817	130 (63,268)	126 (57,243)	0.366	214 (99,358)	118 (57,232)	88 (43,181)	167 (108,292)	**<0.001**

Note that our patients were quite similar in most characteristics to the general U.S. 2002 incident hemodialysis population (our cohort vs. U.S.): % aged 65 or older, 39.8% vs. 52.3%; % white: 60.3% vs. 63.5%; % male, 53.6% vs. 54.3%; % diabetes as cause of end-stage renal disease, 47.3 vs. 45.0%; mean body mass index, 27.5 vs. 27.5; mean albumin 3.5 vs. 3.1 g/dl, mean hemoglobin, 9.8 vs. 10.0 g/dl; and mean creatinine, 7.3 vs. 7.1 [28].

ICED = Index of Coexistent Disease, scored on a 3-point scale (0 = no, 1 = mild, 2 = moderate, 3 = severe comorbidity); BMI = body mass index; Ca-P = calcium phosphate; CRP = C-reactive protein; IQR = interquartile range.

*By analysis of variance/*t *test (continuous variables) or χ^2 ^test (categorical variables).

### Association of attainment of targets with mortality

**Figure 1:**
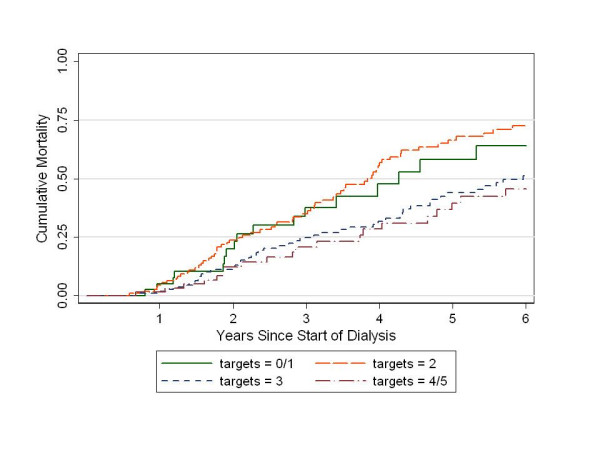
Cumulative mortality by number of KDOQI clinical performance targets attained at 6 months. P = 0.002 by log-rank test.

The crude mortality rates at the median follow-up of 2.8 years were lower in the patients who attained the albumin target and in those who attained multiple targets (Table 2). A Kaplan-Meier survival curve (Fig. 1) suggested that those patients attaining three or more targets may have a survival advantage over those attaining fewer or no targets.

**Table 2:** Association of attainment of 6-month clinical performance targets with mortality

**Target**	% Dead*	**Relative Hazard (95% Confidence Interval)**		
		
		**Unadjusted**	Multivariate Adjusted**	Propensity Score Adjusted***
**Individual Targets**				

Albumin ≥4.0 g/dl (N = 650)				
Target not attained	29.9	1.00 (Ref.)	1.00 (Ref.)	1.00 (Ref.)
Target attained	16.1	**0.49 (0.37–0.65)**	**0.55 (0.41–0.75)**	0.77 (0.55–1.08)
*P*	***<0.001***	***<0.001***	***<0.001***	*0.132*
Ca-P Product <55 mg^2^/dl^2 ^(N = 648)				
Target not attained	24.4	1.00 (Ref.)	1.00 (Ref.)	1.00 (Ref.)
Target attained	26.4	0.83 (0.65–1.04)	**0.74 (0.57–0.95)**	**0.67 (0.52–0.86)**
*P*	*0.574*	*0.109*	***0.020***	***0.002***
Hemoglobin ≥11 g/dl (N = 644)				
Target not attained	29.3	1.00 (Ref.)	1.00 (Ref.)	1.00 (Ref.)
Target attained	23.2	**0.75 (0.60–0.96)**	**0.62 (0.48–0.79)**	**0.67 (0.52–0.87)**
*P*	*0.083*	***0.020***	***<0.001***	***0.002***
Dialysis Dose, Kt/V≥1.2 (N = 539)				
Target not attained	30.5	1.00 (Ref.)	1.00 (Ref.)	1.00 (Ref.)
Target attained	24.8	0.73 (0.51–1.05)	**0.67 (0.45–1.00)**	**0.56 (0.35–0.88)**
*P*	*0.245*	*0.089*	***0.049***	***0.012***
Access Type, fistula (N = 424)				
Target not attained	24.5	1.00 (Ref.)	1.00 (Ref.)	1.00 (Ref.)
Target attained	20.0	0.80 (0.57–1.11)	0.96 (0.67–1.36)	1.05 (0.73–1.52)
*P*	*0.328*	*0.189*	*0.806*	*0.776*

**Albumin, Ca-P, and Hemoglobin Targets (N = 643)**				

0 targets attained	29.9	1.00 (Ref.)	1.00 (Ref.)	1.00 (Ref.)
1 target attained	28.7	0.81 (0.56–1.16)	0.77 (0.52–1.13)	**0.65 (0.44–0.95)**
2 targets attained	25.7	**0.61 (0.41–0.89)**	**0.51 (0.34–0.77)**	**0.49 (0.32–0.73)**
3 targets attained	12.9	**0.32 (0.19–0.53)**	**0.27 (0.15–0.47)**	**0.26 (0.15–0.46)**
*P for trend*	***0.009***	***<0.001***	***<0.001***	***<0.001***

**All Five Targets (N = 344)**				

0–1 targets attained	27.5	1.00 (Ref.)	1.00 (Ref.)	1.00 (Ref.)
2 targets attained	29.3	0.93 (0.52–1.63)	0.90 (0.50–1.63)	0.93 (0.52–1.67)
3 targets attained	20.5	**0.51 (0.28–0.91)**	**0.49 (0.27–0.91)**	**0.53 (0.29–0.97)**
4–5 targets attained	15.5	**0.37 (0.19–0.72)**	**0.33 (0.16–0.63)**	**0.41 (0.20–0.83)**
*P for trend*	***0.030***	***<0.001***	***<0.001***	***<0.001***

Mean at-risk follow-up was 3.3 years (median, 2.8 years). Statistically significant values are in boldface type.

*At median follow-up (2.8 years). *P *by Pearson's χ^2 ^test (individual targets) or nonparametric trend test (multiple targets).

**Adjusted for first value of target(s) indicated, age, race, index of co-existent disease, and albumin.

***Propensity score models consisted of:

Albumin target: age, sex, index of co-existent disease, baseline albumin, creatinine, and C-reactive protein;

Ca-P target: age, sex, employment, index of co-existent disease, creatinine, hemoglobin, and baseline Ca-P;

Hemoglobin target: age, sex, race, body mass index, and baseline hemoglobin;

Dialysis dose target: age, sex, race, body mass index, Kt/V, creatinine, hemoglobin, and Ca-P;

Access target: age, sex, race, index of co-existent disease, albumin, creatinine, and C-reactive protein;

Albumin, Ca-P, and hemoglobin targets: age, sex, index of co-existent disease, body mass index, baseline albumin, baseline hemoglobin, baseline Ca-P, and C-reactive protein; and

All five targets: age, sex, index of co-existent disease, body mass index, baseline Kt/V, baseline albumin, baseline hemoglobin, baseline Ca-P, and C-reactive protein.

In adjusted models, the attainment of all individual targets was associated with a decreased risk of mortality (Table 2). These associations were statistically significant for all individual targets except access type with usual multivariable adjustment. Only albumin and dialysis dose targets remained statistically significantly associated with decreased mortality using the stricter Bonferroni-adjusted threshold. Propensity score adjustment also showed decreased risk of mortality with the attainment of each target except access type, but the association of the albumin target with mortality was no longer statistically significant. Excluding those patients who had already attained the individual target at baseline (giving sample sizes of *n *= 558, 159, 517, 244, and 356 for albumin, Ca-P product, hemoglobin, dialysis dose, and access type, respectively), only albumin remained statistically significant as a predictor of decreased mortality (data not shown). By likelihood ratio tests in multivariable-adjusted models, albumin and hemoglobin (*P *< 0.001 for both) and Ca-P product (*P *= 0.039) contributed most to the variation in mortality. Dialysis dose (*P *= 0.052) and access (*P *= 0.806) did not significantly contribute to variation in mortality.

Attaining one or more of the laboratory targets was associated with decreased risk of mortality (Table 2). When all five targets were examined, only the attainment of three or more targets was associated with decreased risk mortality, but there was again a strong statistically significant trend of decreased risk with each additional target attained (Table 2). In subpopulation analyses of those attaining 0 or 1 targets at baseline, risk of mortality decreased with additional 6-month targets attained, but the trend was not significant (P = 0.201). When only those who attained zero targets at baseline were included (n = 29), results were not significant.

### Association of attainment of targets with hospitalization

#### Incidence of hospitalization

All individual targets were associated with a decreased incidence of hospitalizations, with the exception of attaining the dialysis dose target, which was only statistically significantly associated with decreased hospitalizations with propensity score adjustment (Table 3). Additionally, all targets except dialysis dose remained statistically significantly associated with decreased hospitalization incidence using the Bonferroni-adjusted significance threshold. Excluding those patients who had already attained the individual target at baseline, all individual targets remained statistically significantly associated with decreased hospital admissions, except for the Ca-P product target (*P *= 0.091; *n *= 154). By likelihood ratio test, albumin, Ca-P, hemoglobin and access (*P *< 0.001 for all) all contributed significantly to the variation in hospital admission rate, whereas dialysis dose (*P *= 0.903) did not.

**Table 3:** Association of attainment of 6-month clinical performance targets with hospital admissions and hospital days

**Target**	Average per follow-up year*	**Incidence Rate Ratio (95% Confidence Interval)**		
		
		**Unadjusted**	Multivariate Adjusted**	Propensity Score Adjusted***
**NUMBER OF HOSPITALIZATIONS**				

**Individual Targets**				

Albumin ≥4.0 g/dl (N = 650)				
Target not attained	2.2	1.00 (Ref.)	1.00 (Ref.)	1.00 (Ref.)
Target attained	1.4	**0.62 (0.57–0.67)**	**0.67 (0.62–0.73)**	**0.81 (0.73–0.89)**
*P*	***<0.001***	***<0.001***	***0.001***	***<0.001***
Ca-P Product <55 mg^2^/dl^2 ^(N = 648)				
Target not attained	2.1	1.00 (Ref.)	1.00 (Ref.)	1.00 (Ref.)
Target attained	1.9	**0.88 (0.82–0.95)**	**0.86 (0.79–0.92)**	**0.80 (0.74–0.86)**
*P*	*0.374*	*0.001*	***<0.001***	***<0.001***
Hemoglobin ≥11 g/dl (N = 644)				
Target not attained	2.1	1.00 (Ref.)	1.00 (Ref.)	1.00 (Ref.)
Target attained	1.8	**0.85 (0.79–0.91)**	**0.82 (0.76–0.88)**	**0.81 (0.75–0.87)**
*P*	*0.146*	***<0.001***	***<0.001***	***<0.001***
Dialysis Dose, Kt/V≥1.2 (N = 539)				
Target not attained	1.9	1.00 (Ref.)	1.00 (Ref.)	1.00 (Ref.)
Target attained	1.9	1.00 (0.89–1.13)	0.99 (0.88–1.12)	**0.86 (0.75–0.99)**
*P*	*0.796*	*0.970*	*0.902*	***0.040***
Access Type, fistula (N = 424)				
Target not attained	2.0	1.00 (Ref.)	1.00 (Ref.)	1.00 (Ref.)
Target attained	1.4	**0.73 (0.66–0.81)**	**0.79 (0.71–0.88)**	**0.86 (0.77–0.97)**
*P*	***0.016***	***<0.001***	***<0.001***	***0.011***

**Albumin, Ca-P, and Hemoglobin Targets (N = 643)**				

0 targets attained	2.6	1.00 (Ref.)	1.00 (Ref.)	1.00 (Ref.)
1 target attained	2.1	**0.69 (0.61–0.76)**	**0.69 (0.62–0.78)**	**0.59 (0.53–0.66)**
2 targets attained	1.8	**0.62 (0.56–0.70)**	**0.62 (0.55–0.69)**	**0.57 (0.51–0.65)**
3 targets attained	1.3	**0.43 (0.37–0.50)**	**0.44 (0.38–0.51)**	**0.39 (0.33–0.46)**
*P for trend*	***<0.001***	***<0.001***	***<0.001***	***<0.001***

**All Five Targets (N = 344)**				

0–1 targets attained	2.4	1.00 (Ref.)	1.00 (Ref.)	1.00 (Ref.)
2 targets attained	2.3	0.96 (0.82–1.14)	0.88 (0.74–1.05)	1.01 (0.85–1.19)
3 targets attained	1.7	**0.71 (0.60–0.84)**	**0.67 (0.57–0.80)**	**0.76 (0.64–0.90)**
4–5 targets attained	1.1	**0.55 (0.45–0.66)**	**0.55 (0.43–0.67)**	**0.65 (0.53–0.80)**
*P for trend*	***<0.001***	***<0.001***	***<0.001***	***<0.001***

**DAYS HOSPITALIZED**				

**Individual Targets**				

Albumin ≥4.0 g/dl (N = 647)				
Target not attained	13.0	1.00 (Ref.)	1.00 (Ref.)	1.00 (Ref.)
Target attained	7.4	**0.55 (0.53–0.57)**	**0.61 (0.58–0.63)**	**0.76 (0.72–0.79)**
*P*	***<0.001***	***<0.001***	***<0.001***	***<0.001***
Ca-P Product <55 mg^2^/dl^2 ^(N = 645)				
Target not attained	11.6	1.00 (Ref.)	1.00 (Ref.)	1.00 (Ref.)
Target attained	11.1	**0.89 (0.87–0.92)**	**0.85 (0.83–0.88)**	**0.79 (0.77–0.82)**
*P*	*0.719*	***<0.001***	***<0.001***	***<0.001***
Hemoglobin ≥11 g/dl (N = 641)				
Target not attained	11.7	1.00 (Ref.)	1.00 (Ref.)	1.00 (Ref.)
Target attained	11.0	**0.92 (0.90–0.95)**	**0.89 (0.86–0.92)**	**0.89 (0.86–0.91)**
*P*	*0.571*	***<0.001***	***<0.001***	***<0.001***
Dialysis Dose, Kt/V≥1.2 (N = 537)				
Target not attained	9.3	1.00 (Ref.)	1.00 (Ref.)	1.00 (Ref.)
Target attained	11.6	**1.12 (1.06–1.18)**	**1.11 (1.05–1.17)**	**0.89 (0.84–0.95)**
*P*	*0.225*	***<0.001***	***<0.001***	***<0.001***
Access Type, fistula (N = 420)				
Target not attained	12.3	1.00 (Ref.)	1.00 (Ref.)	1.00 (Ref.)
Target attained	7.3	**0.65 (0.62–0.67)**	**0.72 (0.69–0.75)**	**0.79 (0.75–0.82)**
*P*	***0.004***	***<0.001***	***<0.001***	***<0.001***

**Albumin, Ca-P, and Hemoglobin Targets (N = 640)**				

0 targets attained	13.9	1.00 (Ref.)	1.00 (Ref.)	1.00 (Ref.)
1 target attained	11.7	**0.69 (0.66–0.72)**	**0.70 (0.67–0.73)**	**0.61 (0.59–0.64)**
2 targets attained	11.9	**0.70 (0.67–0.73)**	**0.70 (0.66–0.73)**	**0.67 (0.63–0.70)**
3 targets attained	6.0	**0.37 (0.34–0.39)**	**0.37 (0.35–0.40)**	**0.35 (0.33–0.38)**
*P for trend*	***0.001***	***<0.001***	***<0.001***	***<0.001***

**All Five Targets (N = 341)**				

0–1 targets attained	13.2	1.00 (Ref.)	1.00 (Ref.)	1.00 (Ref.)
2 targets attained	14.3	0.95 (0.89–1.01)	**0.87 (0.82–0.94)**	1.00 (0.94–1.07)
3 targets attained	10.4	**0.71 (0.66–0.75)**	**0.67 (0.63–0.72)**	**0.77 (0.72–0.83)**
4–5 targets attained	4.8	**0.41 (0.38–0.44)**	**0.43 (0.40–0.47)**	**0.50 (0.46–0.55)**
*P for trend*	***<0.001***	***<0.001***	***<0.001***	***<0.001***

For hospital admissions, mean at-risk follow-up (excluding days in the hospital when patients could not be admitted) was 3.1 years (median, 3.0 years); for hospital days, mean at-risk follow-up was 3.2 years (median, 3.1 years). Statistically significant values are in boldface type.

*P by *t *test (individual targets) or nonparametric trend test (multiple targets).

**Adjusted for first value of target(s) indicated, age, race, index of co-existent disease, and albumin.

***Propensity score models consisted of:

Albumin target: age, sex, index of co-existent disease, baseline albumin, creatinine, and C-reactive protein;

Ca-P target: age, sex, employment, index of co-existent disease, creatinine, hemoglobin, and baseline Ca-P;

Hemoglobin target: age, sex, race, body mass index, and baseline hemoglobin;

Dialysis dose target: age, sex, race, body mass index, Kt/V, creatinine, hemoglobin, and Ca-P;

Access target: age, sex, race, index of co-existent disease, albumin, creatinine, and C-reactive protein;

Albumin, Ca-P, and hemoglobin targets: age, sex, index of co-existent disease, body mass index, baseline albumin, baseline hemoglobin, baseline Ca-P, and C-reactive protein; and

All five targets: age, sex, index of co-existent disease, body mass index, baseline Kt/V, baseline albumin, baseline hemoglobin, baseline Ca-P, and C-reactive protein.

Each increase in number of targets attained among the laboratory targets was associated with a statistically significantly decreased incidence of hospitalization (Table 3). There was also a highly significant trend toward decreased incidence with more targets attained. When all five targets were examined, a significant trend of decreased incidence of hospitalization with each additional target attained was seen (Table 3). Only the attainments of three or more targets were individually associated with significantly decreased hospitalization incidence, relative to attaining one or none of the targets. In subpopulation analyses of those attaining 0 or 1 targets at baseline, hospital admissions decreased even more strikingly with each additional 6-month target attained [IRR = 2 targets, 0.70 (0.55–0.89); 3 targets, 0.51 (0.40–0.65); 4 or 5 targets, 0.28 (0.16–0.50); P for trend<0.001]. With only those patients who did not attain any targets at baseline (*n *= 23), results were not significant (*P *= 0.435). Again, history of cardiovascular disease was significantly associated with hospital admissions, but adjusting for it did not change our results. Less frequent physician contact, less frequent sit-down rounds, and noncompliance were all associated with increased hospital admissions but did not affect our estimates of the association of targets with hospital admissions when they were added as adjusters.

#### Days hospitalized

All individual targets except dialysis dose were associated with a decreased hospital days (Table 3), but propensity score adjustment showed a statistically significant decrease in hospital days with the attainment of the dialysis dose target. Additionally, all individual targets remained statistically significantly associated with decreased hospital days using the Bonferroni-adjusted significance threshold. Excluding those patients who had already attained the individual target at baseline, all individual targets remained statistically significantly associated with decreased hospital days (*P *< 0.001 for all). By likelihood ratio test, all five individual targets (*P *< 0.001 for all) all contributed significantly to the variation in number of days hospitalized.

Each increase in number of targets attained among the laboratory targets was associated with decreased hospitalized days (Table 3), and there was a significant trend toward fewer days with more targets attained. The same statistically significant trend of decreased hospital days with each additional target attained was seen when all five targets were examined (Table 3). Subpopulation analyses of those attaining 0 or 1 targets at baseline showed similar results to hospital admissions [IRR = 2 targets, 0.68 (0.62–0.74); 3 targets, 0.55 (0.50–0.61); 4 or 5 targets, 0.15 (0.11–0.20); P for trend<0.001]. Only including those patients who did not attain any targets at baseline (*n *= 23) still gave a significant trend (*P *= 0.030) toward decreased hospital days with more targets attained at 6 months.

#### Hospitalization costs

The attainment of the albumin target was associated with a reduction of approximately $3000 per patient-year in Medicare hospital payments (Table 4) with multivariable adjustment. This target remained statistically significantly with a reduction in payment using the Bonferroni-adjusted significance threshold. With the use of propensity score adjustment, decreased payments were seen with all individual targets, but without statistical significance (Table 4). By likelihood ratio test, albumin (*P *= 0.002) and access (*P *= 0.039) contributed to variation in hospital costs, but Ca-P product (*P *= 0.977), hemoglobin (*P *= 0.679), and dialysis dose (*P *= 0.535) did not.

**Table 4:** Association of attainment of 6-month clinical performance targets with hospital payments

**Target**	**Average Cost per Follow-up Year**		
	
	Crude*	**Unadjusted**	Multivariate Adjusted**
**Individual Targets**			

Albumin ≥4.0 g/dl (N = 470)			
Target not attained	$14,252	$14,333	$9,838
Target attained	$9,406	$9,259	$6,556
*P*	***0.001***	***0.001***	***0.002***
Ca-P Product <55 mg^2^/dl^2 ^(N = 469)			
Target not attained	$13,325	$13,086	$11,586
Target attained	$12,469	$12,608	$11,549
*P*	*0.553*	*0.747*	*0.978*
Hemoglobin ≥11 g/dl (N = 466)			
Target not attained	$13,163	$12,833	$8,511
Target attained	$12,456	$12,659	$8,125
*P*	*0.621*	*0.906*	*0.691*
Dialysis Dose, Kt/V≥1.2 (N = 390)			
Target not attained	$13,774	$14,589	$9,126
Target attained	$12,290	$12,160	$8,155
*P*	*0.495*	*0.300*	*0.553*
Access Type, fistula (N = 308)			
Target not attained	$13,148	$13,384	$13,923
Target attained	$10,111	$9,538	$9,936
*P*	*0.109*	***0.044***	*0.052*

**Albumin, Ca-P, and Hemoglobin Targets (N = 465)**			

0 targets attained	$15,535	$15,534	$7,877
1 target attained	$13,980	$13,815	$7,388
2 targets attained	$11,439	$11,228	$5,870
3 targets attained	$10,461	$11,596	$6,235
*P for trend*	***0.007***	*0.060*	*0.088*

**All Five Targets (N = 248)**			

0–1 targets attained	$12,453	$13,285	$3,165
2 targets attained	$15,366	$14,919	$3,670
3 targets attained	$10,389	$10,446	$2,645
4–5 targets attained	$8,654	$8,624	$2,138
*P for trend*	***0.029***	***0.029***	*0.052*

Mean follow-up was 2.8 years (median, 2.7 years). Statistically significant values are in boldface type. Cost per year was log-transformed; averages of log-transformed cost were calculated (either crudely or using linear regression models), and the averages were exponentiated to reflect dollar values.

*P by *t *test (individual targets) or nonparametric trend test (multiple targets).

**Adjusted for first value of target(s) indicated, age, race, index of co-existent disease, and albumin.

Increases in the number of targets attained among the laboratory targets were generally associated with decreased payments (Table 4). Also, there was a trend toward decreased payments with more targets attained. The trend of decreased payments with each additional target attained was also seen when all five targets were examined (Table 4), but statistical significance was borderline in the adjusted model. Propensity score adjustment showed results similar to those obtained with multivariate adjustment (*P *for trend = 0.129 for all five targets). Subpopulation analyses of those attaining 0 or 1 targets at baseline showed that hospital payments decreased with each additional 6-month target attained ($15,835, $12,708, $11,499, and $10,938 for 0 or 1, 2, 3, and 4 or 5 targets, respectively), but the trend was not significant (P = 0.470).

## Discussion

Attainment of clinical performance targets in patients requires considerable time and effort on the part of providers and patients. Resistance to work on quality improvement may in part be due to skepticism that attainment will lead to meaningful improvement in health and other outcomes [2,3]. This national longitudinal study shows that attainment of each of the individual targets is strongly associated with better outcomes, including decreased mortality, fewer hospitalizations, fewer days in the hospital, and lower overall Medicare hospital payments, validating the professional clinical practice guidelines [4]. A few studies of prevalent hemodialysis patients have shown associations of clinical performance target attainment with decreased hospital admissions and related costs [37,38], but this study adds to our knowledge of the effect of clinical performance target attainment in incident hemodialysis patients. Importantly, our results also provide new evidence that each additional target attained results in incremental improvement in these outcomes, regardless of which targets are attained, and provide a comprehensive examination of a variety of performance measures and outcomes.

For example, we found that attainment of each additional target resulted in a mortality risk reduction of approximately 35%, a hospitalization risk reduction of approximately 20%, a reduction in number of days hospitalized of approximately 24%, and a decrease in annual Medicare hospital payments of approximately $762 per patient-year. The results support the idea that efforts to improve clinical performance (*e.g*., through provider education, competition from public reporting, and rewards, such as payment for performance) might reap large benefits in morbidity, mortality, and efficiency for both patients and payers.

It could be argued that our data are historical and no longer relevant in today's quality improvement-oriented atmosphere. However, recent reports from CMS and the USRDS on the clinical performance of hemodialysis patients in the United States show that 91%, 35%, 80%, and 39% of patients attained Kt/V≥1.2, fistula use, hemoglobin ≥ 11 g/dl, and albumin ≥ 4.0 g/dl, respectively, in the last quarter of 2003 [39,40]. These percentages are higher than those seen in this study (82%, 27%, 60%, and 31%, respectively), but clearly there is still room for improvement in the attainment of these targets.

Although this study was observational, the associations we report do meet many of the criteria for causality, including plausibility, temporality, dose-response relationship, and consistency. It is certainly plausible that attaining clinical performance targets would improve patient outcomes, and it is just as plausible that increasing numbers of targets attained in a patient would result in incrementally better outcomes. The use of an incident dialysis cohort, capturing patients at the inception of their dialysis treatment, ensures temporality, as the exposure to provider efforts to meet clinical performance targets for ESRD patients begins with the initiation of kidney replacement therapy. We observed a dose-response relationship, reflected in the graded trend toward better outcomes with more targets attained. Also, our results were consistent across various modeling strategies and consistent with other studies focusing on single static measures (*e.g*., albumin) or outcomes (*e.g*., mortality).

If this assumption of a causal relationship holds, and if we assume that our population is similar to the general U.S. hemodialysis population in the distribution of targets attained and other characteristics, projected outcomes on a population level are that each additional performance target attained could potentially save 20,440 lives, 113,400 hospital admissions, 952,000 hospital days, and $213 million in Medicare hospital payments in a year. These estimates confirm that there is tremendous potential value in patients attaining multiple targets, not only for decreased mortality, as reported previously [21], but in reduced hospitalizations, length of stay, and associated cost.

What does it take to move toward a better level of performance? Many methods have been and are being attempted. First, performance measurement with feedback to providers has produced demonstrable, but modest, gains. Demonstration of explicit links to health outcomes, such as the results we generated, might further help to convince providers of the importance of better clinical performance. Second, providing performance results and evidence of its link to outcome to consumers or patients could stimulate providers to improve [41-43]. Finally, paying providers for better performance is in its infancy but is rapidly gaining momentum [44,45]. These results provide a firmer foundation for the application of the performance measures that we studied to reward providers for attaining or improving good outcomes. To the extent that our data on hospital payments are true, they may also provide a rationale to return a finite portion of insurer savings on hospital care to providers, rather than rewarding high performers at the expense of other providers.

Some possible limitations to this study deserve mention. First, this study was underpowered to determine which particular individual targets or combinations among the five performance targets examined yield the most benefit in terms of each outcome. Second, it should be remembered that this is an observational study and that only the associations between target and outcome can be ascertained, not cause and effect. Despite the fact that some clinical performance targets have been chosen based upon observational data and consensus (and could never be part of an ethical randomized trial), the value of observational data should not be overstated. Because some of the factors that we studied here were not the subject of national guidelines at the time we started our study, guideline-directed therapeutic intent cannot be shown. The hemoglobin, dialysis dose, and vascular access targets were in place in 1997 and have not changed since this time, although they were not yet widely implemented by 1998. The albumin and Ca-P targets used here were not introduced until well after the study period. Third, as with any study of clinical performance targets, the biological markers studied may be influenced by other factors than the quality of providers' care. For example, albumin and hemoglobin levels could be affected in part by the degree of ultrafiltration achieved. Of course, some targets may be more easily modified (*e.g*., hemoglobin through erythropoietin) than others (*e.g*., albumin, which is influenced by patients' nutritional and inflammatory states). Importantly, there may be fixed and possibly unmeasurable patient characteristics that might or might not allow target attainment with exposure to same quality of provider care. Such characteristics might also affect the amount of time needed to attain a goal, which may be more or less than 6 months for some patients and some targets. Finally, despite collection of and adjustment with extensive data on determinants of patient outcomes, selection bias and residual confounding due to lack of data on variables for which we could not account may still exist.

## Conclusion

This study strongly suggests that clinical performance target attainment is associated with better patient outcomes, including decreased hospitalization, hospital days, and associated costs and better survival. Providers treating chronic disease should be reassured that local and national efforts expended to attain incremental change in the number of performance targets reached results in additional benefit to patients.

## Abbreviations

KDOQI, Kidney Disease Outcomes Quality Initiative; ESRD, end-stage renal disease; Ca-P, calcium-phosphate; EQUAL, ESRD Quality; CHOICE, Choices for Healthy Outcomes In Caring for ESRD; CMS, Centers for Medicare & Medicaid Services; USRDS, United States Renal Data System, BMI, body mass index; ICED, Index of Coexistent Disease; CRP, C-reactive protein; ANOVA, analysis of variance.

## Declaration of competing interests

The author(s) declare that they have no competing interests.

## Authors' contributions

LP performed the statistical analyses and drafted the manuscript; NEF participated in the design of the study and helped to draft the manuscript; BGJ assisted in data interpretation and drafting of the manuscript; JHS, NWL, JC, & MJK participated in the design of the study and provided feedback on the manuscript; NRP conceived of the study, participated in its design, assisted in data interpretation, and helped to draft the manuscript. All authors read and approved the final manuscript.

## Pre-publication history

The pre-publication history for this paper can be accessed here:


